# P53 suppresses expression of the 14-3-3gamma oncogene

**DOI:** 10.1186/1471-2407-11-378

**Published:** 2011-08-25

**Authors:** Vijayababu M Radhakrishnan, Charles W Putnam, Wenqing Qi, Jesse D Martinez

**Affiliations:** 1 Arizona Cancer Center, Department of Cellular & Molecular Medicine, University of Arizona, Tucson, Arizona 85724, USA; 2 Department of Pediatrics, Steele Research Center, 1501 N Campbell Ave, Tucson, Arizona 85724, USA; 3 Department of Surgery, 1501 N Campbell ave, Tucson, Arizona 85724, USA; 4 Department of Medicine, 1515 N. Campbell Ave., Tucson, Arizona 85724, USA

**Keywords:** Lung cancer, 14-3-3, p53 mutations, Gene Copy, Transcription Regulation

## Abstract

**Background:**

14-3-3 proteins are a family of highly conserved proteins that are involved in a wide range of cellular processes. Recent evidence indicates that some of these proteins have oncogenic activity and that they may promote tumorigenesis. We previously showed that one of the 14-3-3 family members, 14-3-3gamma, is over expressed in human lung cancers and that it can induce transformation of rodent cells in vitro.

**Methods:**

qRTPCR and Western blot analysis were performed to examine 14-3-3gamma expression in non-small cell lung cancers (NSCLC). Gene copy number was analyzed by qPCR. P53 mutations were detected by direct sequencing and also by western blot. CHIP and yeast one hybrid assays were used to detect p53 binding to 14-3-3gamma promoter.

**Results:**

Quantitative rtPCR results showed that the expression level of 14-3-3gamma was elevated in the majority of NSCLC that we examined which was also consistent with protein expression. Further analysis of the expression pattern of 14-3-3gamma in lung tumors showed a correlation with p53 mutations suggesting that p53 might suppress 14-3-3 gamma expression. Analysis of the gamma promoter sequence revealed the presence of a p53 consensus binding motif and in vitro assays demonstrated that wild-type p53 bound to this motif when activated by ionizing radiation. Deletion of the p53 binding motif eliminated p53's ability to suppress 14-3-3gamma expression.

**Conclusion:**

Increased expression of 14-3-3gamma in lung cancer coincides with loss of functional p53. Hence, we propose that 14-3-3gamma's oncogenic activities cooperate with loss of p53 to promote lung tumorigenesis.

## Background

14-3-3 proteins are present in all eukaryotic organisms that have been examined and are highly conserved between species. The number of proteins in the 14-3-3 family varies with species. However, in mammals, seven isoforms have been identified named as β, γ, ε, σ, ζ, θ and η, and they function by binding other proteins predominantly through phosphorylated serine residues [1,2]. These proteins are highly conserved and are involved in the regulation of a variety of key physiological pathways such as cell cycle progression [3] apoptosis [4] and mitogenic signaling [5]. Binding target proteins enable 14-3-3 family members to regulate the activity of enzymes, control subcellular localization of their targets, or act as scaffolds that promote protein-protein interactions.

14-3-3 proteins were identified as abundant proteins in the brain and were first described to activate neurotransmitter synthesis [6]. Subsequently, they were implicated in a variety of neurological conditions [7] suggesting that they functioned primarily in the brain. However, 14-3-3 protein family members are widely expressed in mammalian tissues and recent evidence suggests that these proteins may also play a role in the development of human cancers. Examination of 14-3-3 protein levels in human tumors including lung [8], prostate [9], breast [10], oral [11], ovarian [12] and pancreatic cancers [13,14] indicate that 14-3-3 protein expression becomes aberrant during tumorigenesis. However, it is unclear if or how these proteins contribute to tumorigenesis.

Of the 14-3-3 proteins linked to cancer, the best studied is 14-3-3σ, which is a transcriptional target of the p53 tumor suppressor. Activation of p53 by DNA damage leads to induction of 14-3-3σ and G2 arrest [3]. Loss of 14-3-3σ also results in defective DNA damage repair [15] and promotes tumorigenesis in breast epithelia [16]. Moreover, down regulation of 14-3-3σ enables primary human epithelial cells to grow indefinitely [17]. Collectively these findings suggest that 14-3-3σ may function as a tumor suppressor and confirm that 14-3-3 gene expression can be regulated by p53.

The role of 14-3-3γ isoform in cancer is less well understood. However, Jin et al. [18] have shown that 14-3-3γ can inhibit transcriptional activity of p53 and we have previously shown that the 14-3-3γ protein is overexpressed in lung cancers and can promote polyploidy [19]. In order to gain insight into the role that 14-3-3γ may have in lung tumorigenesis we examined their expression and the co-occurrence of p53 mutations in lung tumor specimens. We found evidence suggestive of a functional interaction between 14-3-3γ and p53.

## Methods

Frozen human lung tumor specimens and non malignant tissues were obtained from Cooperative Human Tissue Network, Vanderbilt University Medical Center (Nashville, TN). 80 samples were selected based on the tumor type and percentage of tumor cell content (> 70%) and also 21 normal tissues were selected. These studies were evaluated by the University of Arizona Human Subjects Protection Program and judged to be exempt as the specimens are de-identified. The human lung cancer cells, A549, H358 and H322 cells were obtained from American Type Culture Collection (ATCC), USA. The human colorectal cancer cell lines p53^+/+ ^and p53^-/- ^HCT116 were provided by Dr. Bert Vogelstein (The Johns Hopkins University). Anti-p53 and Anti-14-3-3γ antibodies were obtained from Santa Cruz (Santa Cruz, CA). Antibody to β-actin was purchased from Sigma, St Louis, MO. PCR kits were obtained from Invitrogen, USA. First strand cDNA synthesis kit was obtained from Fermentas, USA.

### Real-Time PCR quantitation of mRNA expression for 14-3-3γ

The mRNA expression level was determined by quantitative reverse transcription-PCR on total RNA, using the ABI PRISM 7700 Sequence Detection System. The RNA was isolated using TRIZOL (Invitrogen, USA) and treated with DNase to avoid amplification of genomic DNA. cDNA was synthesized from 500 ng of total RNA and it was used for PCR. PCR was carried out in 50 μl reaction mixture containing 25 μl of SYBR Green qPCR SuperMix-UDG, 1.5 μl of 10 μM each forward and reverse primer, 2 μl of cDNA and water to 50 μl. Thermal cycling conditions were carried at 50°C for 2 min, 95°C for 2 min, 40 cycles of 95°C for 30 sec and 58°C for 30 sec using the ABI PRISM 7700 Sequence Detection System. The primers for 14-3-3γ and GAPDH transcript are listed in Table 1. All experiments were repeated three times. Data are expressed as ΔCt values [ΔCt = Ct of the target gene (14-3-3γ) minus Ct of the GAPDH]. To calculate number of fold changes compared with normal tissues, 2^-ΔΔCt ^equation was used; all statistics were performed with ΔCt values.

**Table 1:** List of primer sequences used for mRNA expression of 14-3-3γ and GAPDH

	Forward (5' > 3')	Reverse (5' > 3')
**14-3-3γ**	CTGAATGAGCCACTGTCGAA	GCACGGACCATCTCAATCTT
**GAPDH**	AGGGCCCTGACAACTCTTTT	AGGGGTCTACATGGCAACTG

### Quantitative-PCR for relative gene quantity of 14-3-3γ

Normal and tumor specimens were embedded in OCT and sections were prepared using a MICROM GmbH cryostat (Waldorf, Germany). The sections were fixed in 75% ethanol and stained in 1.5% eosin for 30 sec. Then they were washed in 95% ethanol, dehydrated in 100% high-grade ethanol, incubated with Xylene, air dried, and subjected to laser capture microdissection (LCM) using the Veritas Microdissection instrument (Arcturus Engineering, CA) and CapSure LCM Caps (Arcturus Engineering, CA). DNA was extracted using the Picopure DNA extraction kit (Arcturus Engineering, CA). Q-PCR was performed using the ABI PRISM 7700 sequence Detection System (Applied Biosystems, USA) by TaqMan based technique. The sequences of the genomic primers and probes used for Q-PCR are listed in Table 2. The primers were tested to ensure amplification of single discrete bands. Relative quantification was performed using the comparative CT (ΔCT) method. To normalize the Q-PCR data against aneuploidy that commonly occurs in NSCLCs [20], the Ct values of the 14-3-3γ gene are normalized with phosphoinositide-3-kinase regulatory subunit 1 (PI3KR1) gene, which is unaltered in NSCLCs [21]. Each real-time qPCR reaction mixture contained 10 μl of DNA sample (obtained from Picopure DNA extraction kit), 12.5 μl of Platinum Quantitative PCR SuperMix-UDG (Invitrogen, USA), 0.75 μl of 10 μM gene-specific forward and reverse primers, 0.25 μl of 10 μM fluorescent probe and water to 25 μl. Thermal cycling conditions were carried at 50°C for 2 min, 95°C for 2 min, 40 cycles of 95°C for 30 sec and 58°C for 30 sec using the ABI PRISM 7700 Sequence Detection System. Samples were run on a 2% agarose gel to ensure that only a single amplicon was produced.

**Table 2:** List of primer sequences used for amplification of 14-3-3γ and PI3KR1 genes by Qpcr

	Forward (5' > 3')	Reverse (5' > 3')	Probe (5' > 3')
**14-3-3γ**	CCTCAGCTGCTCGCTCTG	CGGAGAAGGAGGAGGACACT	6-FAM- CGGTCCTCTCCGGCACTTGGGC-TAMRA
**PI3KR1**	AAGTCTTAAGTTTGGGTTG AGTCG	TAATGATTGACCAAGCTTTTA TGC	6-FAM- TAATGATTGACCAAGCTTTTATGC-TAMRA

### RT-PCR and Direct sequencing for p53 mutations

For the mutational analysis of p53 gene exons 5-9, RT-PCR analysis and direct sequencing were performed. cDNA from tumor samples was synthesized from 500 ng of total RNA and then PCR was performed using following primers, forward 5'-GCC AAG TCT GTG ACT TGC ACG-3' and reverse 5'-AGA GGA GCT GGT GTT GTT GG-3'. The cycling conditions used for these PCRs were as follows: 94°C for 5 min, 30 cycles of 94°C for 30 sec, 55°C for 45 sec and 72°C for 1 min, with a final extension step of 72°C for 10 min. The PCR products were gel purified using gel purification kit (Qiagen, USA). Purified DNA samples were sequenced at the University of Arizona Genetics Core facility.

### Western blot for expression of 14-3-3γ, p53 and β-actin proteins

The protein lysates from either frozen sections or cell lines were collected using ice-cold RIPA buffer containing 150 mM NaCl, 50 mM Tris, 1 mM EDTA, 1% NP-40, 0.5% sodium deoxycholate, 0.1% SDS, pH 7.4 and 2 μl/ml Protease inhibitor cocktail (Sigma, USA). Protein concentrations were determined using the BioRad protein assay kit (Biorad, USA) and 50 μg protein was separated by 12% SDS-PAGE gel. The proteins were then transferred onto a nitrocellulose membrane (Millipore, USA) and then blocking was carried out by incubating with 5% nonfat milk in TBST buffer. After blocking, the membranes were incubated with primary antibodies raised against human 14-3-3γ (Rabbit polyclonal, Santa Cruz, USA), p53 (DO1, mouse monoclonal, Santa Cruz, USA) and β-actin for 1 hr. Then the membranes were washed three times, incubated with secondary-HRP (Sigma, USA) for 1 hr and then washed with TBST buffer for three times. Blots were developed by Super Signal West Pico detection system (Pierce, Rockford, IL). The membranes were stripped and reprobed with β-actin monoclonal antibody (Sigma, USA) to confirm equal loading. For frozen tumor specimens, the protein quantification from western blot image was done with the help of Image J program (NIH, MD) and 2-fold above the normal is considered as 14-3-3γ overexpressing tumors.

### One Hybrid Assays

The Grow'n'Glow GFP One-Hybrid System (MoBiTec, USA) was used to test for p53 binding to YWHAG promoter sequences. The prey control plasmid pJG4-5-p53, supplied with the kit, consists of the p53 cDNA fused to the B42 activation domain; expression of the p53 fusion protein is under the control of the GAL1 promoter. The 600 and 1200 bp promoter sequences were subcloned into the GFP reporter vector ("bait" plasmid), pGNG2, which contains the GFP_UV _gene driven by the GAL1, 10 minimal promoter. The 600 and 1200 bp fragments were amplified by PCR from the pGL3 plasmid containing complete 14-3-3γ promoter using the following primers: 600 bp forward primer, 5'-GAGAGCGGCCGCGTCGGTCCTCTCCGGCACTT-3'; 1200 bp forward primer, 5'-GAGAGCGGCCGCATGAACGAGAATATATCAGCGTGACC-3'; reverse primer for both reactions, 5'-GAGAACTAGTCTTCGCGGGGCTGGGTCT-3'. A NotI recognition sequence is incorporated into the forward primers; the reverse primer includes a SpeI recognition sequence. The amplification products and the pGNG2 plasmid were cut with NotI and SpeI, and the promoter sequences were ligated into the vector. The two promoter constructs were verified by sequencing. The p53 encoding plasmid was co-transformed with reporter construct into *Saccharomyces cerevisiae *strain, W303 and successful transformants selected on -ura -trp synthetic medium containing dextrose. Individual colonies were inoculated into -ura -trp SC medium with 2% dextrose and grown to mid-log phase. The cells were then split in two, washed with sterile water, and resuspended in -ura -trp SC medium containing either 2% sucrose or 2% galactose. After six hours, cell density was measured spectrophotometrically (OD_600_) and 200 μL aliquots loaded onto a 96-well microplate. The plates were inspected visually under long wave UV light for green fluorescence; the fluorescence was quantified using a plate reader (excitation at 395 nm and emission at 509 nm) (Molecular Devices, USA). The mean values for each promoter construct and carbon source were normalized for cell density and expressed as the ratio of fluorescence in galactose to that in sucrose, the latter set at one fold.

### ChIP assays

We collected A549, H358, H322, HCT116p53^+/+ ^and HCT116p53^-/- ^cells for ChIP assay 8h after γ-irradiation. ChIP assays were carried out essentially as described [22]. p53 immunoprecipitation was done with 5 μg of antibody against p53 (DO1, Santa Cruz, USA), or with Mouse IgG (Santa Cruz, USA) as a negative control. We carried out PCR amplification using primers (forward, 5'-AACCACTGTGGCCAGCCGGTAT-3'; reverse, 5'-ACAGGAGGCGCGTCCATTGT-3'), designed to give a product including the p53-binding element. The PCR protocol was 30 cycles of a 45 sec denaturation step at 94°C, a 1 min annealing step at 58°C and a 1-min extension step at 72°C. The PCR products were resolved by 1.5% agarose gel electrophoresis.

### Plasmid Constructions

To create the pGL3-100, pGL3-586, pGL3-830, pGL3-840, pGL3-850 and pGL3-1200 14-3-3γ promoter plasmids, the genomic DNA from human foreskin fibroblast cells (HFF1 cells, provided by Dr. Rilo, University of Arizona) was PCR amplified with forward primers hanging KpnI site and reverse primers hanging NheI site, products were digested and gel purified followed by ligation with KpnI/NheI digested pGL3 linearized vector. For PGL3-1200 Δ850-840 and PGL3-1200 Δ850-830, 350 bp fragment upstream of p53 RE (-830 to -850) was amplified by PCR with forward primer hanging KpnI. 840 and 830 bp downstream from p53RE products were amplified with reverse primers hanging NheI site. These products were ligated together with KpnI/NheI digested PGL3 empty vector followed by transformation. All the promoter reporter constructs were sequenced and confirmed at the University of Arizona sequencing facility.

### Transfections and Luciferase Assays

All of the transfections were done in triplicate in 24-well plates. Approximately, 1 × 10^3 ^cells/well were seeded 24 h before transfection. Plasmids were transfected into cells using Lipofectamine reagent (Life Technologies, Inc.). Luciferase assays were performed using the Dual Luciferase Assay System (Promega, USA) that already contains an internal control detectable simultaneously with the luciferase reporter gene. Each experiment was conducted at least in triplicates. Ad-GFP and Ad-P53-GFP adenoviruses are laboratory stocks. Cells were at 60% confluence when infected with 10MOI. siRNA duplexes targeting the p53 mRNA was chemically synthesized by Dharmacon Research. Their target sequences are as follows: p53, 5'-CAGTCTACCTCCCGCCATA-3' (p53 siRNA-1) and 5'-GAAGAAACCACTGGATGGA-3' (p53 siRNA-2). The control siRNAs is as follows: 5'-GGCTACGTCCAGGAGCGCACC-3'.

### Statistical analysis

The statistical analysis was performed by analysis of variance. Only ΔCt values were used for the statistical analysis of gene amplification and mRNA expression. The Dunnett's multiple comparison was used to test the statistical significance between normal tissues and tumor tissues. Pearson correlation was used to correlate fold changes of gene amplification and mRNA followed by Student's t-test.

## Results

### Increased levels of 14-3-3γ mRNA in lung tumors

**Figure 1:**
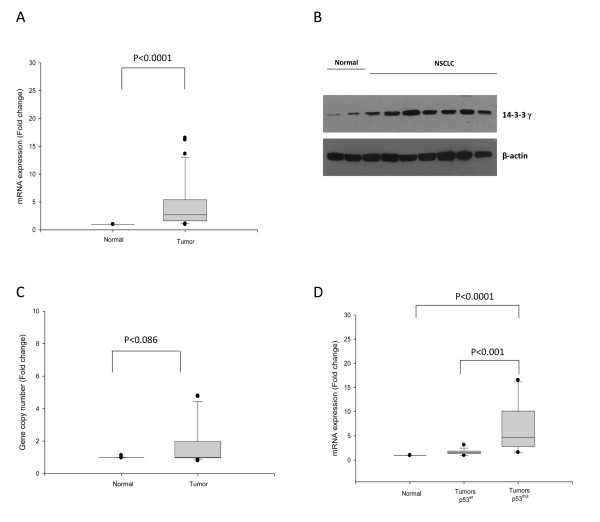
**Elevated expression of 14-3-3γ correlates with mutations in p53**. *A*. *mRNA expression in NSCLC tumors*. RNA was extracted from frozen specimens of normal and tumor tissues and the quantity of 14-3-3γ determined using quantitative RT-PCR. Fold changes were determined by 2^-ΔΔCt ^by comparing Ct values of 14-3-3γ with GAPDH. The box plot depicts 2^-ΔΔCt ^values for 14-3-3γ expression. p-values are for comparison of the levels of 14-3-3γ mRNA in tumor specimens with normal tissue, which is calculated using ΔCt values. *B. Analysis of 14-3-3γ expression by Western blotting in NSCLC tumors*. A Western blot showing expression of 14-3-3γ protein in 8 representative NSCLC tumors and protein loading was confirmed by β-actin. *C. Gene copy number*. Genomic DNA was extracted from normal and tumor tissues and the relative quantity of 14-3-3γ DNA determined using quantitative PCR from equal quantities of DNA. ΔCt values for 14-3-3γ were normalized against PI3KR1 (p85alpha regulatory subunit of PI3K) since the latter was found to be unaltered in NSCLCs. The fold changes were calculated by comparing normal and tumor tissues using 2^-ΔΔCt^. Y-axis shows the fold change comparing normal and tumor tissues. *D. 14-3-3γ expression and p53 mutations in NSCLC tumors*. p53 mutational status was determined in the same lung tumors used in panels A and B using RT-PCR amplification of p53 mRNA followed by direct sequencing. Tumors were then segregated based on p53 mutation status, (wt-p53) or (mut-p53) and the 14-3-3γ mRNA expression determined as in panel A. p-values were determined by comparing normal tissue, tumors with wt-p53 and tumors with mut-p53.

We previously showed using qualitative PCR and immunohistochemistry that transcript levels of six of the seven different 14-3-3 genes were elevated in human non-small cell lung cancer (NSCLC) [8]. We showed that one of these, 14-3-3γ, acts as an oncogene in focus formation assays suggesting that it might play a role in lung tumorigenesis [23]. In order to gain insight into the mechanism that resulted in the elevated expression in lung tumors, we first utilized quantitative rtPCR to quantify the relative expression levels and to confirm that the expression of 14-3-3γ was transcriptionally elevated. We examined expression levels of 14-3-3γ in 80 frozen samples of non small cell lung cancer (NSCLC) obtained by surgical resection through the Cooperative Human Tissue Network. Total RNA was extracted from the frozen tissues; cDNA was synthesized, and subjected to quantitative RT-PCR. The levels of 14-3-3γ mRNA transcript was significantly increased (p < 0.0001), by an average of six fold when compared to normal tissues (Figure 1A). Western blotting conducted with a subset of the tumors also confirmed that the levels of 14-3-3γ was elevated (Figure 1B). Hence, the elevated levels of mRNA also resulted in elevated protein levels of 14-3-3γ.

### Overexpression of 14-3-3γ does not result from gene amplification

Since our initial data indicated that 14-3-3γ was overexpressed in lung cancers, we next sought to determine the cause of the elevated mRNA levels. One potential explanation is that the 14-3-3γ gene may be amplified. Consequently, we determined the relative quantity of 14-3-3γ DNA using quantitative PCR and compared the values with values obtained from normal tissue. As can be seen in Figure 1C, the quantity of 14-3-3γ gene was altered in some of the tumors, but overall the data did not show a significant change when compared with the normal lung tissue specimens (p < 0.086). Since the gene quantity data was derived from the same set of tumors as the expression data, we also tested for a correlation between increased gene quantity and increased mRNA levels. As expected, we did not find a correlation between 14-3-3γ gene amplification and overexpression of 14-3-3γ mRNA (p value: 0.336; Pearson correlation coefficient: 0.085). Therefore, the elevated levels of 14-3-3γ mRNA could not be attributed to an increase in gene copy number.

### Relationship between 14-3-3γ overexpression and p53 mutational status

The observation by Hermeking and coworkers [3] showing that p53 and 14-3-3 are functionally related prompted us to determine whether expression of 14-3-3γ was influenced by p53 mutational status. To test this, we determined the p53 mutational status in our bank of tumors as described in methods and compared this with 14-3-3γ mRNA expression levels. When we compared 14-3-3γ expression with the occurrence of p53 mutations (Figure 1D), we found a significant correlation between elevated 14-3-3γ RNA levels and mutations in p53 suggesting a functional interaction between p53 mutations and increased 14-3-3γ expression. The 14-3-3γ protein expression in lung tumors was also significantly correlated with p53 mutations (p < 0.0001, Table 3). We also tested for a correlation between p53 mutations and 14-3-3γ gene amplification. We found no significant difference when we compared 14-3-3γ gene amplification with p53 mutational status (data not shown). Therefore, elevated 14-3-3γ gene expression occurs in tumors which have mutations in p53.

**Table 3:** Expression of 14-3-3γ correlated with mutant p53

	No. cancer tissues	14-3-3γ expression (> 2-fold, %)	*P*
**Total**	80	49 (61.2)	
**P53 wild-type**	35	33 (67.3)	0.0001
**P53 mutant**	45	16 (32.7)	

### Repression of 14-3-3γ mRNA and protein expression by wt-p53

**Figure 2:**
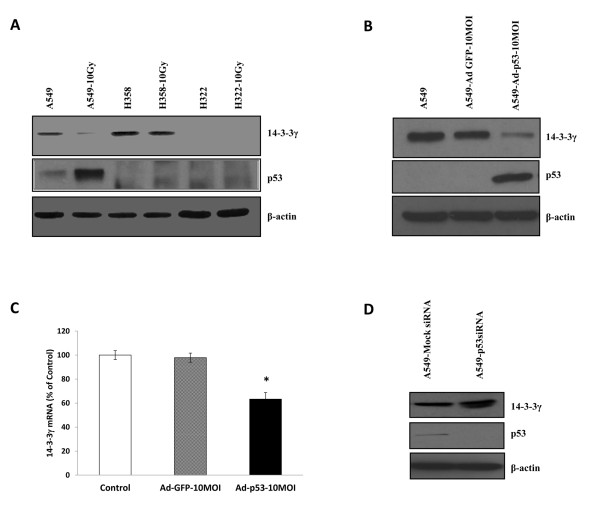
**Wt-p53 suppresses expression of 14-3-3γ**. *A. Expression of 14-3-3γ and p53 after γ-irradiation*. Cultures of A549, H358 and H322 cells were either left untreated (A549, H358, H322) or exposed to 10 Gy of gamma radiation (A549-10 Gy, H358-10 Gy, H322-10 Gy). Total cell extracts prepared six hours after γ-irradiation and proteins fractionated on SDS-PAGE gels. Protein blots were probed for the presence of 14-3-3γ and p53 using the appropriate antibodies. The β-actin was used as a loading control. Typical results are shown. *B. 14-3-3γ protein expression following Ad-p53 *infection. A549 cells were grown and then either left untreated (A549) or infected at a multiplicity of infection (MOI) of 10 with a control adenovirus expressing GFP (A549-AdGFP) or with an adenovirus expressing a wt-p53-GFP (A549-Adp53). Seventy two hours after infection the cells were harvested and the presence of 14-3-3γ and p53 detected in total cell proteins by immunoblotting using the appropriate antibodies. β-actin was used as a loading control. *C. 14-3-3γ mRNA expression following Ad-p53 *infection. The relative levels of 14-3-3γ mRNA expression in samples of panel B were quantified using real-time RT-PCR. The bars depict % change compared with control samples from three different experiments ± SD. Bars showing a significant reduction in message level are marked with an asterisk (p < 0.01 versus Control or Ad-GFP infected). *D*. *14-3-3γ protein expression following endogenous p53 knockdown by siRNA*. A549 cells were cultured and then transfected either with a control non-targeting siRNA (A549-Mock siRNA) or an anti-p53 siRNA (A549-p53siRNA) as described in Materials and Methods. The cells were harvested after 48 h, extracts prepared, and protein blots probed for 14-3-3γ and p53 using the appropriate antibodies. β-actin was used as a loading control.

Our observations in human lung tumors suggested that wt-p53 might suppress 14-3-3γ expression. To explore this further we began by testing whether activation of p53 using ionizing radiation had any effect on 14-3-3γ. We exposed three cell lines, A549 (wt-p53), H358 (no p53 expression) and H322 (mut-p53) to ionizing radiation and then examined the levels of 14-3-3γ expression. As shown in Figure 2A, strong induction of p53 levels was associated with a decrease in the expression of 14-3-3γ in response to γ-irradiation. In contrast, no induction of p53 protein was observed in either H358 or H322 cells. The same trend was observed in γ-irradiated HCT116 p53^+/+ ^and HCT116 p53^-/- ^cells (data not shown), suggesting that 14-3-3γ expression is regulated by p53. To confirm that the reduced 14-3-3γ expression was due to p53 and not an indirect effect of ionizing radiation, we examined 14-3-3γ mRNA and protein levels after infection of A549 cells with a p53 expressing adenovirus. 14-3-3γ protein levels were consistently decreased after transduction with wild-type Adp53 but not with the GFP-adenovirus control (Figure 2B). Consistent with this, qRT-PCR showed that 14-3-3γ mRNA was decreased almost 30% in cells infected with the wt-p53-adenovirus when compared with the GFP-adenovirus control (Figure 2C). Conversely, suppression of p53 expression in A549 cells using siRNA led to increased 14-3-3γ protein expression (Figure 2D). These experiments indicated that p53 could suppress the expression of 14-3-3γ.

### Human 14-3-3γ gene contains a putative p53 consensus binding element

**Figure 3:**
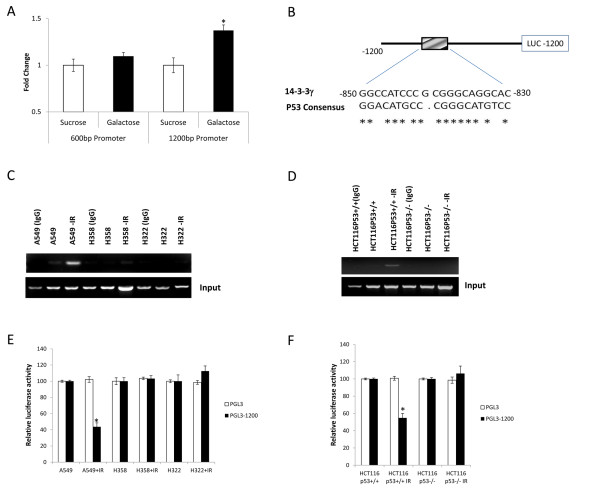
**p53 binds 14-3-3γ promoter and negatively regulates 14-3-3γ expression**. *A. Yeast one hybrid assays*. The 600 and 1200 bp 14-3-3γ promoter sequences were subcloned into the GFP reporter vector as described in materials and methods. The p53 encoding plasmid was co-transformed with reporter vectors into *Saccharomyces cerevisiae *strain W303 and successful transformants were used for the GFP fluorescent assay in the presence of UV. The results were normalized to cells grown in the sucrose medium. Values in y-axis are represented as fold change. Experiments were repeated three times and p values calculated using student's t-test (*p < 0.05). *B*. *A schematic diagram of the 1200 bp of the human 14-3-3γ gene promoter is shown with the position and sequence of the putative p53 binding motif identified*. The p53 consensus binding motif is shown for comparison. Asterisks denote identity between the two sequences. This promoter fragment was cloned into PGL3 adjacent to the luciferase gene (shown at right). *C. CHIP assays in lung cancer cell lines*. A549, H358 and H322 cells were either left untreated (A549, H358, H322) or exposed to 10 Gy ionizing radiation (A549-IR, H358-IR, H322-IR). The cells were harvested eight hours after γ-irradiation and chromatin immunoprecipitation performed as described in the methods section. Reactions were performed either with a mouse IgG as a negative control (A549 -p53ab, H358 -p53ab, H322 -p53ab) or the anti-p53 DO1 antibody. PCR products were separated on agarose gels and visualized by staining with ethidium bromide and viewing with UV illumination. Three independent experiments were performed. Typical results are shown. *D. CHIP assays in human colon cancer cell lines*. HCT116p53^+/+ ^or HCT116p53^-/- ^cells were either left untreated or exposed to 10 Gy ionizing radiation and chromatin immunoprecipitation reactions performed as in panel C using either a mouse IgG as a negative control (-p53ab) or with the anti-p53 DO1 antibody. PCR products were visualized as in panel C. Reactions using input DNA are shown at the bottom of the panel. Three independent experiments were performed. A typical result is shown. *E. 14-3-3γ promoter reporter assays in lung cancer cell lines following γ-irradiation*. A549, H322, or H358 cells were transiently transfected either with empty vector (open bars) or with the reporter depicted in panel B (solid bars). Forty eight hours after transfection the cells were either left untreated (A549, H358, H322) or exposed to ionizing radiation (10 Gy). The cells were harvested eight hours after gamma-irradiation and luciferase activity measured and normalized relative to renilla luciferase to control for variations in transfection efficiency. The bars represent the average of three experiments ± SD. An asterisk denotes those values that are significantly reduced relative to unirradiated controls (*p < 0.01; control vs IR). *F*. *14-3-3γ promoter reporter assays in colon cancer cell lines following γ-irradiation*. HCT116p53^+/+ ^or HCT116p53^-/- ^cells were treated in the same way as cells in panel E. Luciferase activity was determined in the non-irradiated and irradiated (IR) cells as described above. The bars depict the average of three independent experiments, ± SD. Open bars show values for cells transfected with the empty vector. Solid bars show values for cells transfected with the reporter vector shown in panel B. An asterisk marks those values that were significantly reduced relative to non-irradiated controls (*p < 0.01; control vs IR).

Modulation of 14-3-3γ expression by p53 could be either indirect, that is, by inducing another protein which in turn might regulate 14-3-3γ expression, or direct, by p53 itself binding to the promoter region of 14-3-3γ. To determine whether or not p53 could bind to the promoter region of 14-3-3γ we utilized the GFP_UV _one-hybrid system, using as "bait" 600 or 1200 bp fragments of the promoter region 5' to the start codon and as "prey", mouse p53. Under long wave UV light, budding yeast co-transformed with bait and prey plasmids exhibited strong green fluorescence with 1200 bp promoter fragment. Quantification of GFP emission revealed that the 1200 bp fragment produced a significant GFP signal when compared with sucrose control which indicated that p53 could bind with the 14-3-3γ promoter (Figure 3A).

p53 protein is a transcription factor that specifically recognizes and binds to DNA consensus sequences defined as PuPuPuC(A/T) (T/A)GPyPyPy (N)_0-14 _PuPuPuC(A/T) (T/A)GPyPyPy, in which Pu stands for purine, Py stands for pyrimidine, and N stands for any nucleotide. Analysis of the human 14-3-3γ promoter sequence using the TF search program revealed the presence of a putative p53 consensus binding element in the promoter (Figure 3B). To determine if p53 would indeed bind to the putative p53 consensus binding elements in human 14-3-3γ gene in vivo, ChIP assays were performed in A549, H322, H358, HCT116 p53^+/+ ^and HCT116 p53^-/- ^cells that were either untreated or exposed to 10 Gy gamma radiation. We found evidence of p53 binding to the response element in cells that expressed a wt-p53 and that this effect was enhanced with exposure to ionizing radiation. Little or no binding was observed in cells that expressed no or mutant p53 (Figures 3C &3D).

### Endogenous wt-p53 inhibited the promoter activity of 14-3-3γ

To confirm that p53 could suppress 14-3-3γ expression we cloned 1200 bp of the 14-3-3γ promoter and inserted it into the pGL3 luciferase reporter plasmid and transfected this into A549, H322 or H358 cells which were either left untreated or exposed to gamma radiation. Consistent with our CHIP assays, we found that reporter activity was suppressed by as much as ~50% when the cells were exposed to radiation, but that reporter activity was not affected in cells that expressed no or a mutant p53 (Figures 3E). The repression of 14-3-3γ promoter activity by ectopic expression of wt-p53 suggested that endogenous p53 could inhibit or modulate 14-3-3γ promoter activity. To test whether the p53 repression of 14-3-3γ is a physiologically relevant response we examined the ability of endogenous p53 in A549 cells to repress 14-3-3γ promoter activity in response to gamma radiation, a known potent p53 inducer [24]. We reasoned that if 14-3-3γ is a p53 target gene for repression, the 14-3-3γ promoter activity should be reduced after p53 induction in cells containing wt-p53 (Figure 3E). Indeed, we found that 14-3-3γ promoter activity was reduced in irradiated A549 cells, but not in either H358 or H322 cells. To further test this we also examined reporter activity in HCT116p53^+/+ ^and HCT116p53^-/- ^cells. Consistent with our results in A549 cells we found that reporter activity was reduced in irradiated HCT116p53^+/+ ^but not in HCT116p53^-/- ^cells or non-irradiated HCT116p53^+/+ ^(Figure 3F). These data strongly supported the notion that 14-3-3γ gene expression is negatively regulated by p53.

### The -850 to -830 region is sufficient to mediate p53 repression activity

**Figure 4:**
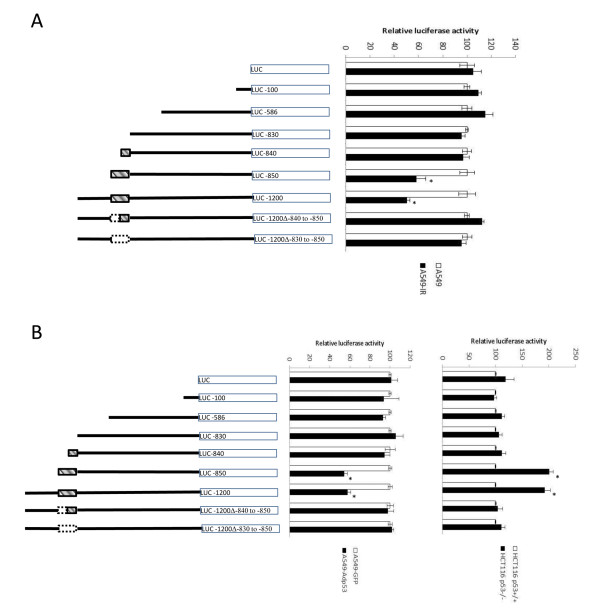
**Deletion of the p53 binding motif in the 14-3-3γ promoter eliminates suppression by p53**. A series of deletion mutants were constructed that successively deleted segments from the 5' end of the 14-3-3γ promoter. These are illustrated diagrammatically linked to a luciferase gene and are shown on the left side of panels A and B. The p53 binding motif is represented as a small striped box. Small boxes outlined with stippled lines show the position of p53 consensus sequences that were deleted. The numerical designation shown within the luciferase box indicates the length of the 14-3-3γ promoter sequence attached to the luciferase gene. *A. Endogenous p53 repress wild-type 14-3-3γ promoter activity after γ-irradiation*. Luciferase reporter vectors each containing either no 14-3-3γ promoter sequences (PGL3-LUC), 14-3-3γ promoter sequences of various lengths (PGL3-100 bp-LUC, PGL3-586 bp-LUC, PGL3-830 bp-LUC, PGL3-1200 bp-LUC), or 14-3-3γ promoter sequences that lacked either all or part of the p53 consensus binding sequence (PGL3-840 bp-LUC, PGL3-850 bp-LUC, PGL3-1200Δ850-840 or PGL3-1200Δ850-830) were transiently transfected into A549 cells together with a renilla luciferase vector. Forty-eight hours after transfection the cells were either left untreated (open bars) or irradiated with 10 GY ionizing radiation (solid bars). The cells were harvested 12 hours later, extracts prepared and luciferase activity quantified. The bars in the graph at the right of panel A represent the mean ± SD of three independent experiments. Values that were determined to be significantly reduced relative to the matched control are marked with an asterisk (p < 0.01; control vs IR). *B*. *14-3-3γ wild-type and mutant promoter reporter assays in A549 and HCT116 cells*. The 14-3-3γ promoter deletion reporter vectors described in panel A were transfected into A549 cells that are infected with either GFP-expressing adenovirus (open bars) or with an adenovirus expressing a wt-p53 (solid bars). Forty-eight hours later the cells were harvested and luciferase activity quantified and normalized to renilla luciferase activity. Bars in the graph in the middle of panel B represent the mean of three independent experiments ± SD. Values that were found to be significantly reduced relative to the matched control are marked with an asterisk (p < 0.01; Ad-GFP vs Ad-p53). In a similar experiment, HCT116p53^+/+ ^(open bars) and HCT116p53^-/- ^(closed bars) with the same vectors described above and irradiated with 10 Gy ionizing radiation. Luciferase activity was determined and normalized to renilla luciferase and the values graphed. Results shown represent the mean of three independent experiments ± SD. Values determined to be significantly reduced relative to their matched controls are marked with an asterisk (p < 0.01; HCT116 p53^+/+ ^vs HCT116 p53^-/-^).

Finally, we constructed a set of promoter deletion mutants to test whether p53's negative regulatory activities were mediated through the putative consensus binding site. A 1200 bp stretch of DNA 5' of the 14-3-3γ gene was cloned and deletions that removed successively larger regions from the 5' end of the sequence were generated and linked to a luciferase reporter. In addition, we constructed deletions promoter that removed either part or the entire putative p53 DNA binding motif. The deletion constructs are diagramed in Figure 4. To test reporter response to radiation activated p53, A549 cells were transiently transfected with the reporter plasmids and then either exposed to radiation or not and the luciferase activity was measured. As shown in Figure 4A, the sequences between -586 and -830 were required for activity of the 14-3-3γ promoter and that the complete p53 consensus binding site was required for suppression of reporter activity in response to ionizing radiation.

To further test that p53 could directly repress 14-3-3γ, we infected A549 cells with a wt-p53-GFP adenovirus or a control GFP-expressing adenovirus and measured luciferase activity (Figure 4B). As can be seen, 14-3-3γ promoter activity was down regulated in cells that were infected with the wt-p53 adenovirus, but not with the GFP-expressing adenovirus. Moreover, only those reporters that contained the full length p53 binding motif were negatively regulated by p53. To rule out the possibility that this transcriptional regulation resulted from the interference of the endogenous p53 by the p53-expressing adenovirus, we also tested the promoter activity in human colon cell line HCT116p53^+/+ ^and HCT116p53^-/- ^cells. Consistent with our other experiments, activity of the 14-3-3γ promoter with a complete p53 response element was repressed in HCT116p53^+/+ ^cells, but not in HCT116p53^-/- ^cells (Figure 4B). Taken together our results are consistent with the hypothesis that the 14-3-3γ promoter is negatively regulated by binding to a p53 DNA binding motif in the promoter region.

## Discussion

The novel finding of this study is that 14-3-3γ is negatively regulated by p53 by binding to its promoter. Studies with human non small cell lung cancers have shown that expression of 14-3-3γ directly correlated with the p53 status, and elevated protein expression resulted from an increase in the quantity of mRNA, suggesting that there is a functional interaction between elevated 14-3-3γ expression and loss of p53. Although we found some evidence of 14-3-3γ gene amplification in some tumors, there was no significant correlation with elevated levels of gene expression. Hence, gene amplification could not account for up regulation of 14-3-3γ expression. Previously, we showed that 14-3-3γ caused polyploidy in lung cancer cell lines suggesting that elevated levels of expression of this family member may lead to genomic instability [19]. Therefore, it may be that increased 14-3-3γ expression cooperates with loss of p53 in the promotion of genomic instability in lung cancer.

Studies using in vitro experiments showed two lines of evidence suggesting that p53 repression of human 14-3-3γ is a physiologically relevant response. First, endogenous induction of wt-p53by γ-irradiation repressed the expression of 14-3-3γ at the levels of mRNA and protein. Second, ectopic expression of wt-p53 significantly suppressed the expression of 14-3-3γ. Therefore, overexpression of human wt-p53 can exert a strong inhibitory effect on human 14-3-3γ gene expression and tumors having mut-p53 showed strong expression. Despite the lack of studies on the regulation of 14-3-3γ gene expression, our findings suggest that p53 could be one of the regulators, which may, when inactivated, contribute to the elevated level of 14-3-3γ gene expression in tumor tissues. It is interesting to observe that wt-p53 induced repression of the human 14-3-3γ transcription was mediated by direct binding to its promoter. The important observation is that human 14-3-3γ has a p53 binding site and this site is conserved with the known reported p53-repressed genes [25]. Here the binding of p53 to its response element could result in direct repression of 14-3-3γ gene. Interestingly, the other 14-3-3 isoform, 14-3-3σ, which is well studied, is positively regulated by p53 [3]. Even though 14-3-3γ protein shares more than 80% homology in protein sequence with 14-3-3σ, it is negatively regulated by p53. This functional difference between the two proteins is still unclear.

The finding that human 14-3-3γ is subject to p53 repression, as reported here, provides the first linkage between p53, a powerful tumor suppressor, and 14-3-3γ, an oncogene that promotes genomic instability and tumorigenesis. The fact that the 14-3-3γ promoter has a p53 binding site indicates that 14-3-3γ expression is regulated at the transcriptional level by p53.

## Conclusion

In summary, this study has identified 14-3-3γ as a downstream negatively regulated p53 target protein and that loss of p53 function leads to over expression of 14-3-3γ in lung cancer. Our studies may provide the basis for further understanding of the role of 14-3-3γ in lung tumorigenesis and may open up potential targets for therapeutic approaches.

## Competing interests

The authors declare that they have no competing interests.

## Authors' contributions

VMR carried out experimental design, molecular cloning, CHIP assay, Western blot and qPCR. CWP performed yeast hybrid assay. WQ carried out western blot for 14-3-3γ. JDM designed the experiments and analyzed the data. VMR and JDM wrote the manuscript. All the authors have read and approved the final manuscript.

## Pre-publication history

The pre-publication history for this paper can be accessed here:

http://www.biomedcentral.com/1471-2407/11/378/prepub
